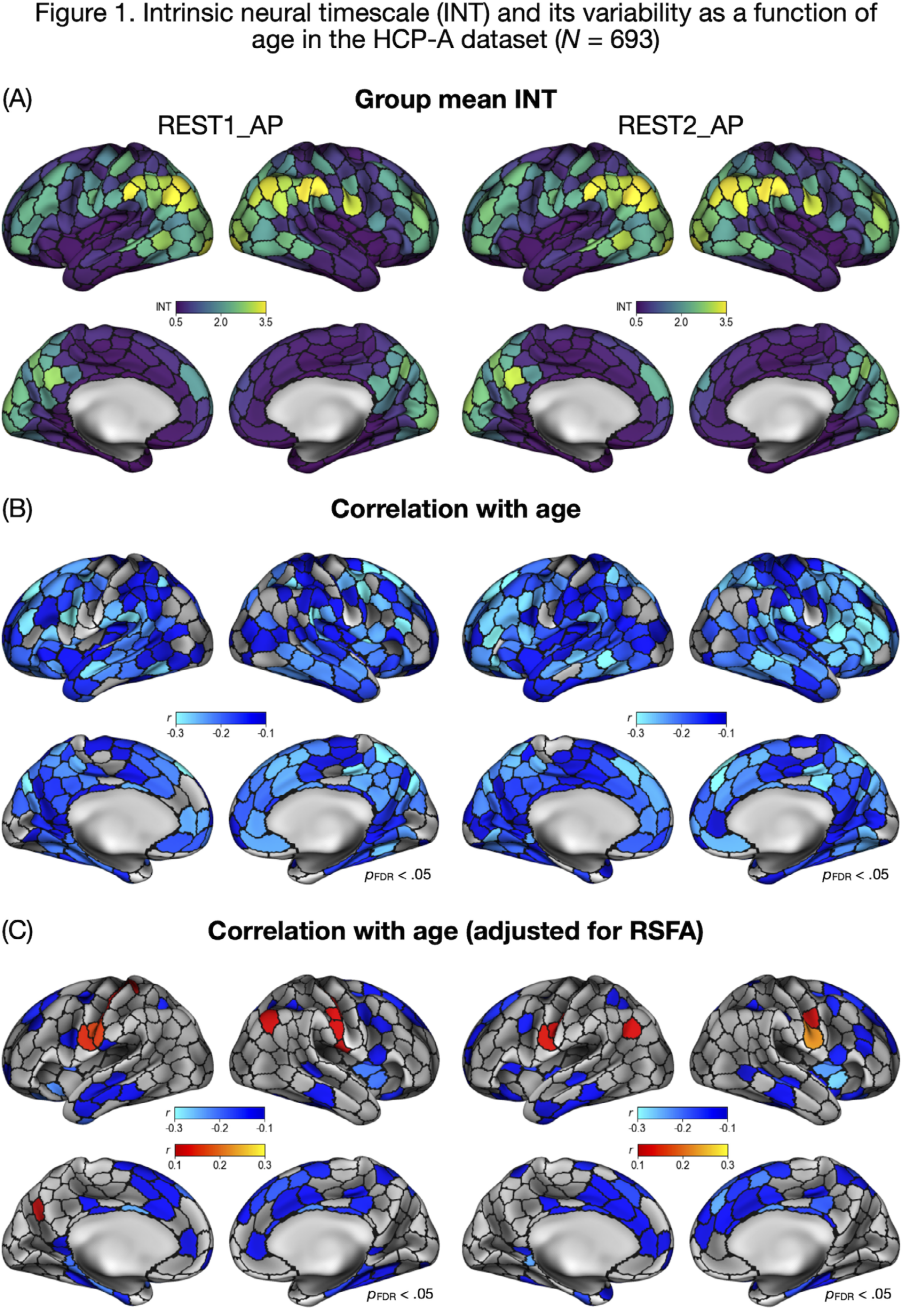# Age‐related variability in BOLD fMRI‐based regional intrinsic neural timescales is influenced by vascular factors

**DOI:** 10.1002/alz70856_106689

**Published:** 2026-01-08

**Authors:** Clare Shaffer, Caitlin C Loxton, Yuta Katsumi

**Affiliations:** ^1^ Northeastern University, Boston, MA, USA; ^2^ Frontotemporal Disorders Unit, Massachusetts General Hospital, Boston, MA, USA; ^3^ Frontotemporal Disorders Unit and Massachusetts Alzheimer's Disease Research Center, Department of Neurology, Massachusetts General Hospital and Harvard Medical School, Boston, MA, USA

## Abstract

**Background:**

Intrinsic neural timescale (INT) refers to the persistence of local neural activity over time and is thought to play a key role in brain functional integration and segregation. While current evidence suggests that INT in some cortical regions is altered in normal aging and Alzheimer's disease, prior studies have also yielded mixed results. This may be due to improper control for cerebro‐ and/or cardiovascular factors, which has been shown in some cases to sufficiently explain age‐relate differences in the temporal variability in blood‐oxygen‐level‐dependent (BOLD) fMRI signal. Here, we examined a large sample of middle‐aged and older participants to test the hypothesis that INT would vary as a function of age, but that the magnitude and topography of age differences would change once BOLD fMRI signal is adjusted for vascular factors.

**Method:**

We analyzed whole‐brain BOLD fMRI data (two runs; 6’30’’ each) collected at wakeful rest from cognitively normal adults as part of the Human Connectome Project‐Aging study (*n* = 693, 394 females, mean age = 60.13 ± 15.53). Using data preprocessing, we estimated INT as temporal autocorrelation decay to a value of .5 (i.e., the time it takes for lagged self‐correlation of BOLD fMRI signal to reach 50% of its maximal value). We additionally quantified resting‐state fluctuation amplitude (RSFA), an established method to normalize BOLD fMRI signal for cerebro‐ and cardiovascular factors across individual participants. Associations between age and INT were examined with and without RSFA as a covariate.

**Result:**

Replicating prior work, we found longer INT in heteromodal association cortical areas (e.g., inferior parietal lobule, posteromedial cortex, lateral frontal cortex) and to some extent visual cortex (Figure 1A). Advancing age was associated with shorter INT in widespread heteromodal and limbic cortical areas (Figure 1B). Notably, once individual variability in vascular factors is accounted for, the relationship between age and INT became weaker and spatially less extensive, with primary involvement of the insula and mid‐cingulate cortex (Figure 1C).

**Conclusion:**

Calibrating BOLD fMRI signal for vascular contributions is essential in examining age differences in INT. Future work based on more direct measures of cerebrovascular reactivity and cardiovascular health is necessary to validate these findings.